# Nitric oxide mediates antimicrobial peptide gene expression by activating eicosanoid signaling

**DOI:** 10.1371/journal.pone.0193282

**Published:** 2018-02-21

**Authors:** Md. Sadekuzzaman, Yonggyun Kim

**Affiliations:** Department of Plant Medicals, Andong National University, Andong, Korea; Institute of Plant Physiology and Ecology Shanghai Institutes for Biological Sciences, CHINA

## Abstract

Nitric oxide (NO) mediates both cellular and humoral immune responses in insects. Its mediation of cellular immune responses uses eicosanoids as a downstream signal. However, the cross-talk with two immune mediators was not known in humoral immune responses. This study focuses on cross-talk between two immune mediators in inducing gene expression of anti-microbial peptides (AMPs) of a lepidopteran insect, *Spodoptera exigua*. Up-regulation of eight AMPs was observed in *S*. *exigua* against bacterial challenge. However, the AMP induction was suppressed by injection of an NO synthase inhibitor, L-NAME, while little expressional change was observed on injecting its enantiomer, D-NAME. The functional association between NO biosynthesis and AMP gene expression was further supported by RNA interference (RNAi) against NO synthase (SeNOS), which suppressed AMP gene expression under the immune challenge. The AMP induction was also mimicked by NO alone because injecting an NO analog, SNAP, without bacterial challenge significantly induced the AMP gene expression. Interestingly, an eicosanoid biosynthesis inhibitor, dexamethasone (DEX), suppressed the NO induction of AMP expression. The inhibitory activity of DEX was reversed by the addition of arachidonic acid, a precursor of eicosanoid biosynthesis. AMP expression of *S*. *exigua* was also controlled by the Toll/IMD signal pathway. The RNAi of Toll receptors or Relish suppressed AMP gene expression by suppressing NO levels and subsequently reducing PLA_2_ enzyme activity. These results suggest that eicosanoids are a downstream signal of NO mediation of AMP expression against bacterial challenge.

## Introduction

Upon microbial pathogenic infection, insects express highly efficient immune responses that are innate and include both humoral and cellular reactions [1]. The humoral responses include hemolymph-clotting activity and phenol oxidase-mediated melanization as well as various antimicrobial peptides that target bacteria and fungi [2–4]. The cellular responses are executed by circulatory hemocytes that participate in phagocytosis, nodulation, and encapsulation depending on the types and numbers of invading pathogens [5]. In addition, insect immunity can exhibit adaptive plasticity by performing immune priming via generating alternative splicing variants of pattern recognition receptors (PRRs) such as the Down syndrome cell adhesion molecule [6].

The highly efficient and complicated insect immune responses are systemically propagated by immune mediators after PRR recognition signals against pathogen-associated molecule patterns [7]. Based on chemical types, four different groups of insect immune mediators have been identified as playing crucial roles in mediating both cellular and humoral responses [8].

The first group is cytokines, small proteins that include Upd (unpaired) molecules in JAK/STAT signaling, Spätzle, Eiger, plasmatocyte-spreading peptide (PSP), and Edin [9]. PSP is expressed in hemocytes and fat body as a proPSP that is activated by proteolytic cleavage to a 23 residue PSP that mediates plasmatocyte-spreading behavior [10]. PSP induces hemocyte-spreading behavior via an approximately 190 kDa receptor [11]. PSP is a member of the ENF peptide family [12], which includes growth-blocking peptide and paralytic peptide. These ENF peptides share a common property of mediating hemocyte-spreading and -aggregation behaviors by altering cytoskeleton rearrangement [13–15]. Silencing PSP expression leads to impaired hemocytic antibacterial activity [16].

The second group of insect immune mediators is the monoamines, including serotonin (= 5-hydroxytryptamine) and octopamine [17,18]. The monoamines enhance hemocyte migration, phagocytosis, and nodulation by altering cell structure via actin-cytoskeleton rearrangement [19,20]. In addition, these monoamines mediate the change of sessile hemocytes into circulatory form by altering adhesiveness to surface via activating the small G protein, Rac1 [21].

The third group is nitric oxide (NO), a small membrane-permeable signal molecule that is synthesized from L-arginine by NO synthase (NOS) [22]; NO mediates both cellular and humoral immune responses in insects [23,24]. NOS expression regulation determines the immune responses of *Manduca sexta*, and variation in the NO levels of different *Drosophila melanogaster* strains reflects their differing susceptibility to pathogenic bacteria [25,26]. In mosquitoes that transmit malarial protozoans, NOS expression is rapidly induced after blood feeding, which elevates NO concentrations [27]; the NO directly limits development of the parasites [28,29].

The fourth group of insect immune mediators is eicosanoids, a group of oxygenated C20 unsaturated fatty acids that mediate both cellular and humoral responses against various pathogens [8]. Eicosanoids include prostaglandin, leukotriene, and epoxyeicosatrienoic acid, and these are usually produced from arachidonic acid (AA: 5,8,11,14-eicosatetraenoic acid) by cyclooxygenase, lipoxygenase, and epoxygenase [30]. AA is rich in phospholipids and released by the catalytic activity of phospholipase A_2_ (PLA_2_) [31]. Upon bacterial challenge, eicosanoids mobilize sessile hemocytes [32] and mediate hemocyte migration to the foci of infections [33]. At the infection sites, eicosanoids mediate phagocytosis [34], nodulation [35], and encapsulation [36] depending on pathogen type. Eicosanoids also mediate antimicrobial peptide (AMP) expression in *Bombyx mori* [37] and *Drosophila melanogaster* [38]. Furthermore, interrupting eicosanoid biosynthesis by inhibiting PLA_2_ activity in the beet armyworm, *Spodoptera exigua*, results in suppressing AMP biosynthesis [39].

There are cross-talks between immune mediators and eicosanoids in which the eicosanoid is the most downstream signal to activate immune responses [8]. PSP and monoamines activate a small G protein, Rac1, which induces PLA_2_ activity to produce eicosanoids in *S*. *exigua* [14]. NO activates hemocyte-spreading behavior and nodule formation, in which an addition of a PLA_2_ inhibitor significantly suppresses the cellular responses of *S*. *exigua* [24]. NO mediates AMP gene expression in two different insects, *M*. *sexta* and *Bombyx mori* [23,25]. This suggests a possibility of NO mediation of AMP gene expression in *S*. *exigua*. Furthermore, the activation of NO on PLA_2_ activity [24] suggests that NO mediates AMP gene expression via eicosanoids.

For this study, we tested a hypothesis that NO mediates AMP gene expression via eicosanoid signal. To test this hypothesis, we used eight different AMP genes that were known to be associated with *S*. *exigua* immune response [39].

## Materials and methods

### 2.1. Insect rearing and bacterial culture

*S*. *exigua* fifth instar larvae (L5) with average body weight of 136.80 ± 16.24 mg were collected from a laboratory colony for experiments. The colony was reared under a constant temperature (25 ± 1°C) on an artificial diet [40]; the adults were fed a 10% sugar solution. *Paenibacillus polymyxa* SC2, *Eschericha coli* BL21, *Xenorhabdus hominickii* ANU101, and *Bacillus thuringiensis aizawai* were cultured in tryptic soy medium (Becton Dickinson, Sparks, MD, USA). *E*. *coli* and *P*. *polymyxa* were cultured at 37°C and 30°C, respectively, overnight in a shaking incubator at 180 rpm. *X*. *hominickii* was cultured at 28°C at 180 rpm shaking overnight. *B*. *thuringiensis aizawai* was cultured at 30°C with 180 rpm shaking for 48 h. For sporulation, the 48 h-cultured bacteria were kept at 4°C for 1 day before the pathogenicity testing.

### 2.2. Chemicals

Arachidonic acid (AA: 5,8,11,14-eicosatetraenoic acid), dexamethasone [DEX: (11β,16α)-9-fluoro-11,17,21-trihydroxy-16-methylpregna-1,4-diene-3], L-NAME (Nω-nitro-L-arginine methyl ester hydrochloride), D-NAME (Nω-nitro-D-arginine methyl ester hydrochloride), and SNAP (S-nitroso-N-acetyl-DL-penicillamine) were purchased from Sigma-Aldrich Korea (Seoul, Korea) and dissolved in dimethylsulfoxide (DMSO). A PLA_2_ substrate, 1-hexadecanoyl-2-(1-pyrenedecanoyl)-*sn*-glycerol-3-phosphatidylcholine, was purchased from Molecular Probes (Eugene, OR, USA).

### 2.3. Immune challenge to induce AMP expression

To check the AMP gene expression pattern, we injected a 1 × 10^5^ colony-forming unit (cfu) of *E*. *coli* or *P*. *polymyxa* or 50 μg of SNAP in a volume of 2 μL. To inspect the effects of NO on AMP production, we injected an NO inhibitor, L-NAME, for treatment and its inactive enantiomer, D-NAME, for control along with 1 × 10^5^ cfu/larva of *E*. *coli*. To analyze the eicosanoid mediation of AMP expression, we injected a PLA_2_ inhibitor, DEX (10 μg/μL), with either *E*. coli or SNAP. At 8 h post-injection (PI), we collected the whole bodies of larvae to extract RNA.

### 2.4. cDNA preparation and RT-qPCR

We extracted total RNA from *S*. *exigua* L5 larvae using Trizol reagent (Life Technologies, Carlsbad, CA, USA). We synthesized cDNA using RT-Premix oligo-dT (5´-CCAGTGAGCAGAGTGACGAGGACTCGAGCTCAAGCT_(16__)_-3´ (Intron Biotechnology, Seoul, Korea) according to the manufacturer’s instructions. For AMP, we conducted reverse transcriptase-polymerase chain reaction (RT-PCR) with 35 cycles at 95°C for 1 min, 52°C for 1 min and 72°C for 1 min after 5 min at 95°C and a final extension at 72°C for 10 min. We quantified the gene expression by RT-qPCR with a StepOnePlus™ Real-Time PCR System (Applied Biosystems, Waltham, MA, USA) following guidelines [41]. We performed the qPCRs in 40 cycles of 95°C for 20 s, 52°C for 30 s, and 72°C for 30 s after an initial 95°C for 10 min. We used a ribosomal gene, RL32, as a reference to normalize target gene expression to compare expression levels under different treatments. We analyzed the mRNA amounts following comparative CT (ΔΔCT) [42].

### 2.5. Bioassay of bacterial pathogenicity

We used two entomopathogenic bacteria in the pathogenic analysis of *S*. *exigua*; for oral pathogenicity, we used *B*. *thuringiensis aizawai*. We applied the bacterial suspension (7.1 × 10^7^ spores/mL) to L5 larvae by diet dipping. After 12 h feeding, we injected 50 μg of L-NAME or D-NAME into the larvae to inhibit NO synthesis. In addition, we injected 50 μg of SNAP or 10 μg of DEX to rescue NO depletion or to inhibit eicosanoid biosynthesis. We injected the control larvae with the solvent (DMSO) used to dilute the chemicals. We graded mortality at 72 h after chemical injection.

To test the pathogenicity of *X*. *hominickii*, we used hemocoelic injection at a dose of 1.4 × 10^5^ cfu/mL; the bacterial infection was accompanied with the chemical treatment described above. Mortality was measured at 72 h after the bacterial challenge. We conducted all treatments three times, and each test used 10 larvae.

### 2.6. RNA interference (RNAi)

We performed RNAi with double-stranded RNA (dsRNA) and prepared the dsRNA using a Megascript RNAi kit following the manufacturer’s protocol (Ambion, Austin, TX, USA). We targeted three genes (*SeNOS*, *SeToll*, *SeRelish*) with RNAi and partially amplified them using T7 promoter sequence-containing gene-specific primers (S1 Table). We performed PCR using L5 larval cDNA with 40 cycles at 94°C for 1 min, 56°C for 1 min, and 72°C for 1 min after an initial denaturing temperature at 94°C for 5 min. We used the PCR product (1 μg) for *in vitro* transcription to make dsRNA with T7 RNA polymerase for 4 h at 37°C. After the DNA and single-stranded RNA were digested for 1 h and subsequently purified, we mixed the resulting dsRNA molecules with Metafectin PRO (Biontex, Planegg, Germany) in 1:1 volume ratio and incubated for 20 min to form liposomes.

To silent target gene expression, we injected 800 ng of dsRNA in 2 μL volume to L5 larvae of *S*. *exigua* L5 larvae with a micro-syringe (Hamilton, Reno, Nevada, USA). We collected larvae at 0, 24, 48, and 72 h PI for RT-qPCR.

### 2.7. Quantifying NO

We indirectly quantified NO by measuring its oxidized form, nitrate (NO^2-^) using the Griess reagent of the Nitrate/Nitrite Colorimetric Assay Kit (Cayman Chemical, Ann Arbor, MI, USA). In brief, we homogenized the whole bodies of *S*. *exigua* in 100 mM phosphate-buffered saline (pH 7.4) with a homogenizer (Ultra-Turrax T8, Ika Laboratory, Funkentstort, Germany). Our measurements used nine larvae for preparing the enzyme samples, and we repeated the treatment with three biological samples. After centrifugation at 14,000 × *g* for 20 min at 4°C, we used the supernatant to measure the nitrate amounts, and we measured the total protein in each sample by Bradford [43] assay. For a standard curve to quantify nitrate concentrations of the samples, we prepared nitrates with final concentrations of 0, 5, 10, 15, 20, 25, 30, and 35 μM in a 200 μL reaction volume. We recorded the absorbance at 540 nm on a microplate reader (SpectraMax^®^ M2, Molecular Devices, Sunnyvale, CA, USA).

### 2.8. PLA_2_ activity measurement assay

PLA_2_ activity measurement followed the method of Radvanyi et al. [44]. Briefly, a total reaction volume (150 μL) consisted of 136.5 μL of 50 mM Tris (pH 7.0), 1.5 μL of 10% bovine serum albumin, 1 μL of CaCl_2_, 10 μL of enzyme source, and 1 μL of pyrene-labeled substrate (10 mM in ethanol). We used a spectrofluorometer (SpectraMAX M2, Molecular Devices, Sunnyvale, CA, USA) to measure the fluorescence intensity at Ex_345_ and Em_398_, and we calculated the enzyme activity by changes in fluorescence/min. We then calculated the specific enzyme activity by dividing the fluorescence change by the protein amount in the reaction (data presented as ΔFLU/min/μg). We determined the protein concentrations in each enzyme source by Bradford [43] assay and conducted each treatment with three biologically independent enzyme preparations using different larval samples.

### 2.9. Statistical analysis

We analyzed each treatment’s means and variance by one-way ANOVA using PROC GLM in the SAS program [45]. We correlated the means with the least square difference (LSD) at Type I error = 0.05.

## Results

### 3.1. NO induces AMP gene expression of *S*. *exigua*

**Fig 1:**
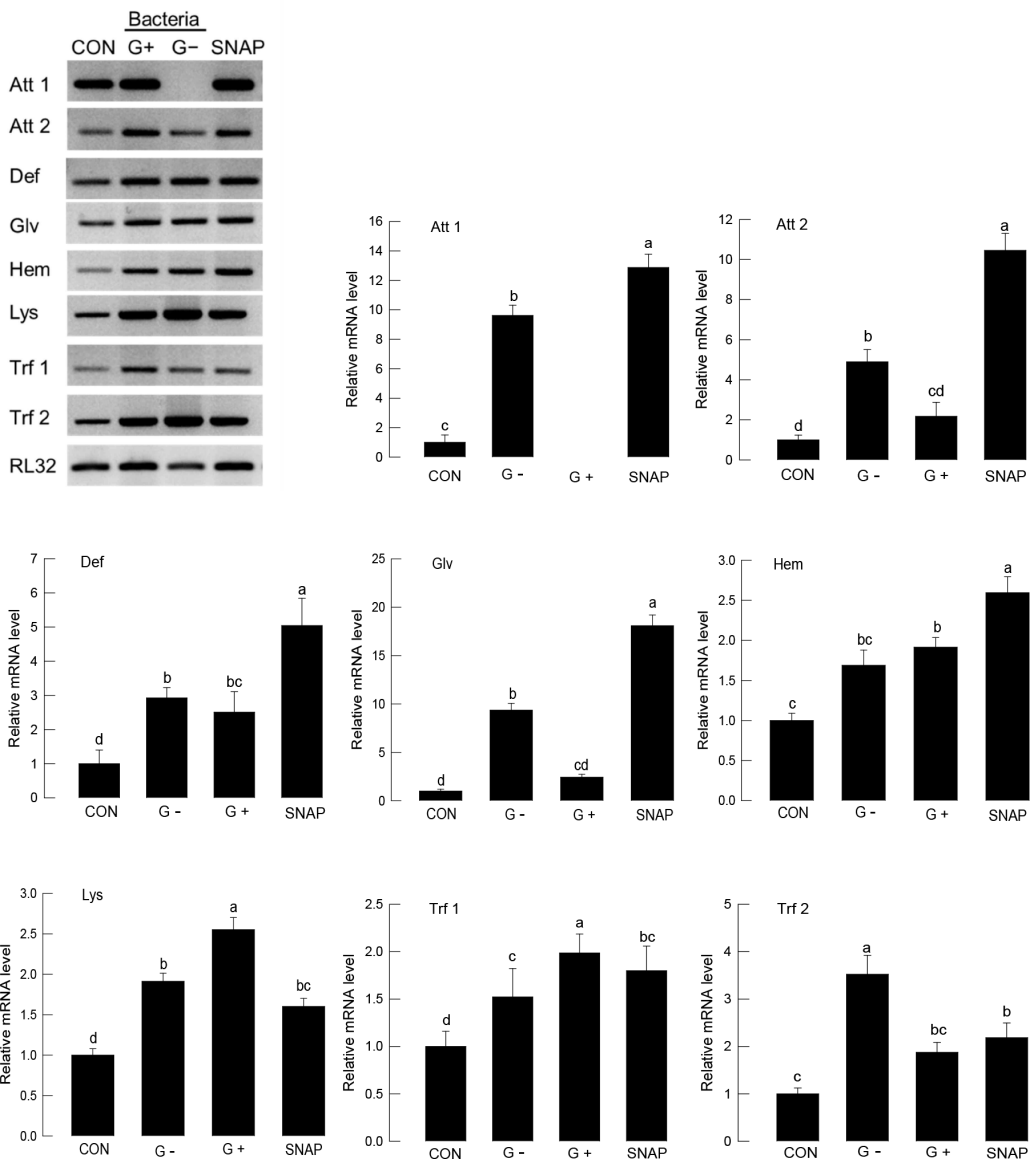
Up-regulation of AMP expression of *S*. *exigua* fifth instar larvae by an NO donor, SNAP. Bacterial challenge used *E*. *coli* for Gram-negative (G-) and *P*. *polymyxa* for Gram-positive (G+) at a dose of 1 × 10^5^ cells per larva. SNAP injection used 50 μg per larva. For control (CON), larvae were injected with a solvent used for dissolving SNAP. After 8 h of injection, each whole body per replication was used for total RNA extraction to prepare cDNA. Each treatment was conducted three times. Expression of eight AMP genes—attacin-1 (Att 1), attacin-2 (Att 2), defensin (Def), gloverin (Glv), hemolin (Hem), lysozyme (Lys), transferrin-1 (Trf 1), transferrin-2 (Trf 2), was quantified by RT-qPCR. RL32, a ribosomal protein, was used as a reference gene for qPCR. Different letters above standard deviation bars indicate significant differences among means at Type I error = 0.05 (LSD test).

Upon bacterial challenge, AMP expression was inducible in *S*. *exigua* (Fig 1). However, the inducible AMP genes were different according to the infected bacterial types. Injecting Gram-negative bacteria (‘G-’) significantly (*P* < 0.05) induced expression of all eight AMP genes. However, Gram-positive bacteria (‘G+’) induced only four AMPs (Def, Hem, Lys, Trf1). Interestingly, all eight AMPs were significantly (*P* < 0.05) induced by injection of SNAP, an NO producer.

**Fig 2:**
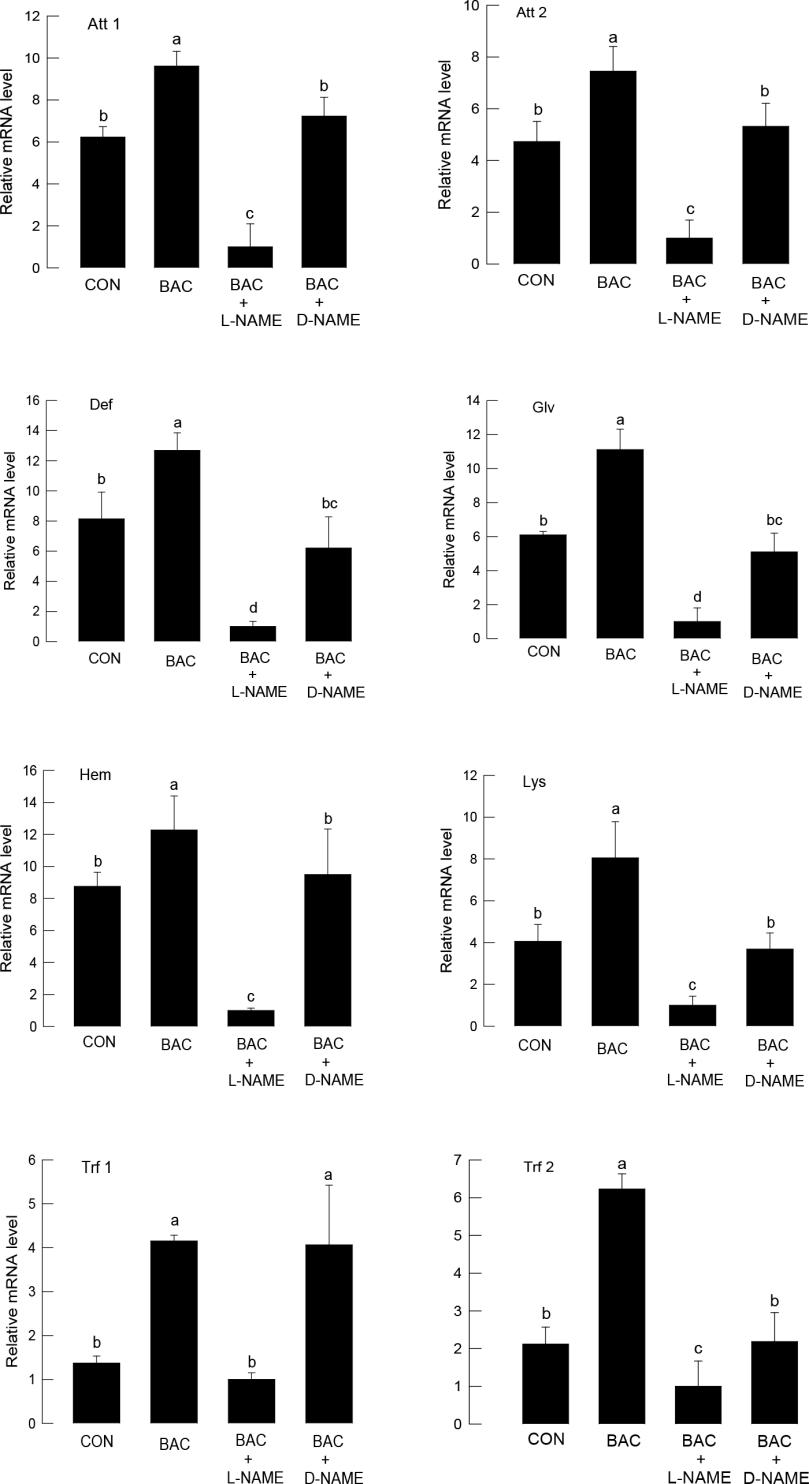
Influence of NO synthase activity on AMP expression of *S*. *exigua* fifth instar larvae. An NO synthase inhibitor, L-NAME, was injected at a dose of 50 μg per larva. D-NAME is its enantiomer and used the same dose. For bacterial challenge (BAC), *E*. *coli* was injected at a dose of 1 × 10^5^ cells per larva. For control (CON), larvae were injected with a solvent used for dissolving SNAP. After 8 h of injection, each whole body per replication was used for total RNA extraction to prepare cDNA; each treatment was conducted three times. Expression of eight AMP genes—attacin-1 (Att 1), attacin-2 (Att 2), defensin (Def), gloverin (Glv), hemolin (Hem), lysozyme (Lys), transferrin-1 (Trf 1), and transferrin-2 (Trf 2), was quantified by RT-qPCR. RL32, a ribosomal protein, was used as a reference gene for qPCR. Different letters above standard deviation bars indicate significant differences among means at Type I error = 0.05 (LSD test).

To further test a hypothesis that AMP expression induced by bacterial challenge was mediated by NO, we injected L-NAME (a specific NOS inhibitor) along with the Gram-negative bacteria (Fig 2). L-NAME significantly (*P* < 0.05) suppressed the induction of gene expression in most AMPs except Trf1. The suppressive activity of L-NAME was sufficiently potent to depress AMP gene expression to levels lower than the control. An enantiomer, D-NAME, also suppressed the AMP gene expressions except that of Trf1. However, it did not inhibit the gene expression as much as L-NAME did.

### 3.2. NO induces AMP gene expressions via eicosanoids

**Fig 3:**
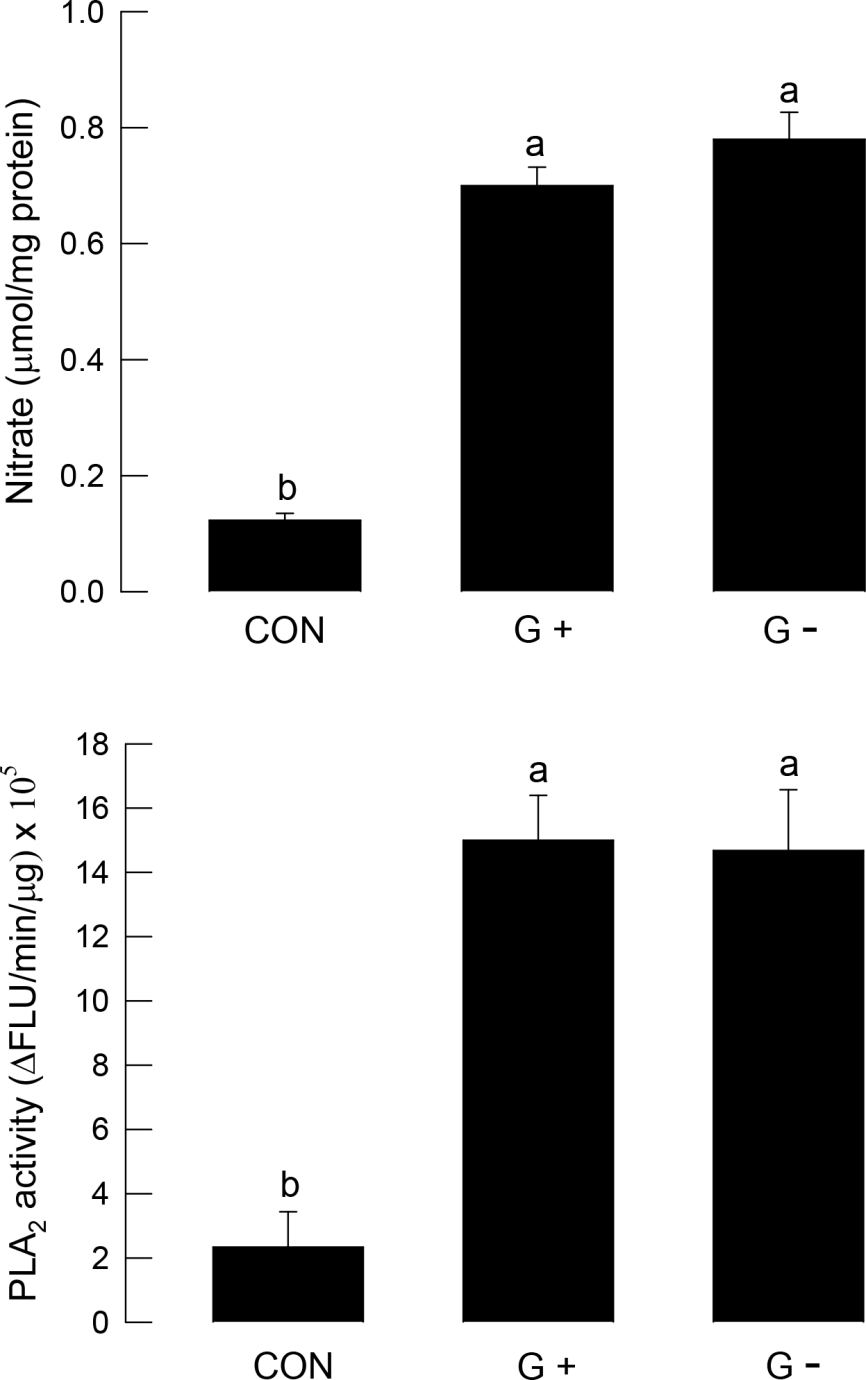
Inducing NO and PLA_2_ activity by bacterial challenge in *S*. *exigua* fifth instar larvae. Bacterial challenge used *E*. *coli* for Gram-negative (G-) and *P*. *polymyxa* for Gram-positive (G+) at a dose of 1 × 10^5^ cells per larva. For control (CON), larvae were injected with a phosphate buffer used for diluting bacterial cells. After 8 h of bacterial infection, the fat bodies were collected and used to assess NO amounts and for PLA_2_ enzyme assay. NO concentration was indirectly measured by quantifying nitrate amount using Griess reagent. PLA_2_ activity was measured using a pyrene-labeled fluorescence substrate. Each treatment was conducted three times. Different letters above standard deviation bars indicate significant differences among means at Type I error = 0.05 (LSD test).

Bacterial challenge significantly (*P* < 0.05) increased NO in larval fat bodies (Fig 3), and the bacterial treatment also up-regulated PLA_2_ activity. There was a positive correlation between NO level and PLA_2_ activity (r = 0.9569; *P* < 0.0001).

**Fig 4:**
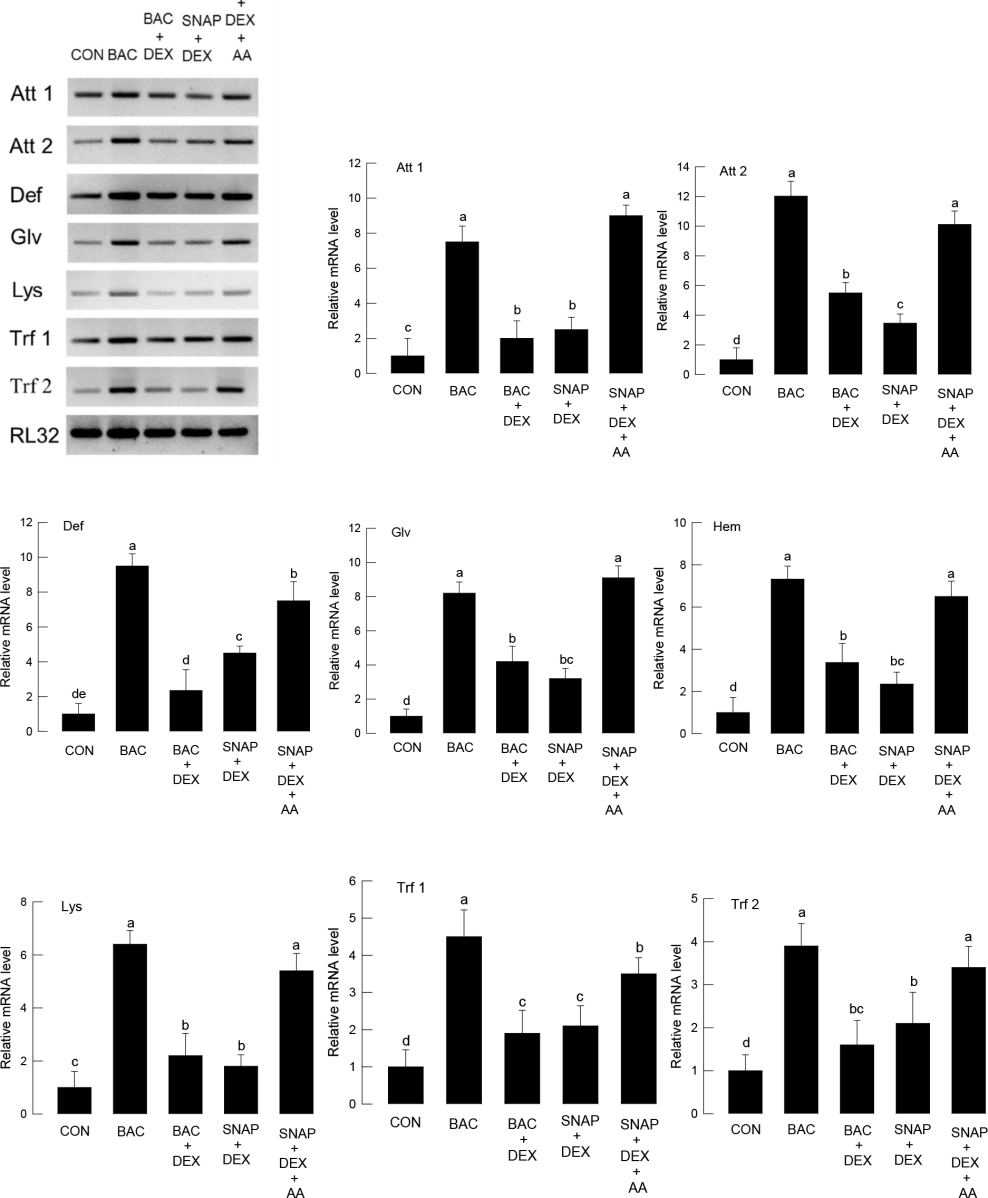
Interaction of NO and eicosanoids in AMP expression of *S*. *exigua* fifth instar larvae. For bacterial challenge (BAC), *E*. *coli* was injected in a dose of 1 × 10^5^ cells per larva. For control (CON), larvae were injected with solvent used for dissolving chemicals. SNAP (an NO donor) injection used 50 μg per larva. Dexamethasone (DEX, a PLA_2_ inhibitor) injection used 10 μg per larva. Arachidonic acid (AA, a PLA_2_ catalytic product) injection used 10 μg per larva. After 8 h of injection, each whole body per replication was used for total RNA extraction to prepare cDNA. Each treatment was conducted three times. Expression of eight AMP genes—attacin-1 (Att 1), attacin-2 (Att 2), defensin (Def), gloverin (Glv), hemolin (Hem), lysozyme (Lys), transferrin-1 (Trf 1), and transferrin-2 (Trf 2), was quantified by RT-qPCR. RL32, a ribosomal protein, was used as a reference gene for qPCR. Different letters above standard deviation bars indicate significant differences among means at Type I error = 0.05 (LSD test).

We further functionally assessed the correlation between NO level and PLA_2_ activity after bacterial challenge with respect to controlling AMP expression (Fig 4). Treatment of a specific inhibitor (DEX) to PLA_2_ suppressed AMP gene expression after Gram-negative bacterial challenge in all eight AMPs. DEX also suppressed the inducible effects of SNAP on AMP gene expression. However, adding AA (a catalytic product of PLA_2_) significantly (*P* < 0.05) rescued the suppressed expressions of all eight AMPs.

**Fig 5:**
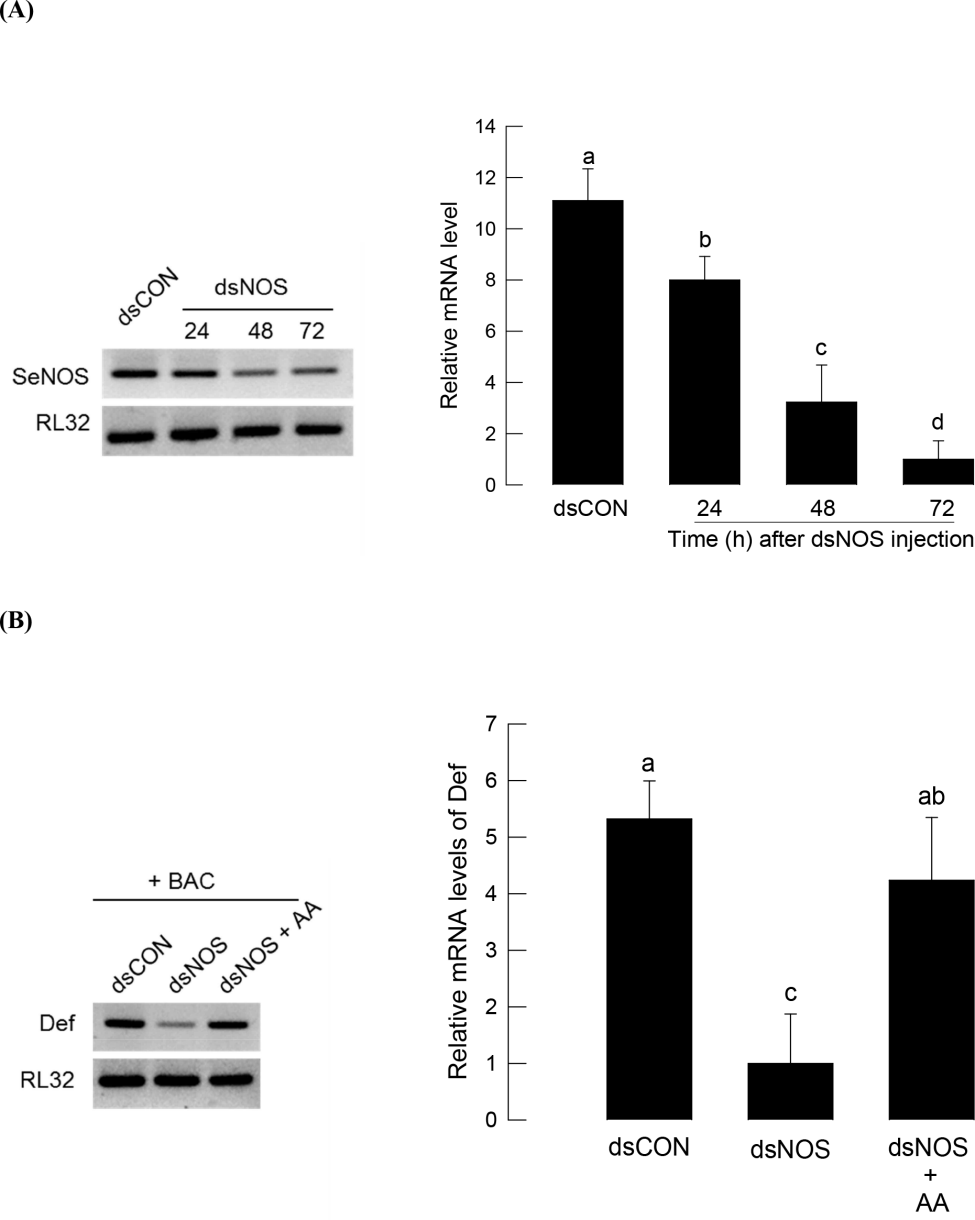
Rescue effect of arachidonic acid (AA, a PLA_2_ catalytic product) on suppressing AMP expression of *S*. *exigua* fifth instar larvae under blocking NO biosynthesis. RNA interference (RNAi) applied to SeNOS using its specific dsRNA at a dose of 800 ng per larva. (A) RNAi effect on *SeNOS* expression. After 24, 48, and 72 h of dsNOS injection, whole bodies were collected to extract RNA and used for cDNA preparation. For RNAi control (dsCON), larvae were injected with dsRNA that were specific to a viral gene, *CpBV-ORF302*, in same doses. (B) Effects of SeNOS RNAi on *defensin* (*Def*) expression. For bacterial challenge (BAC), *E*. *coli* was injected at a dose of 1 × 10^5^ cells per larva after 48 h of dsNOS injection. AA injection used 10 μg per larva. After 8 h of injection, each whole body per replication was used for total RNA extraction to prepare cDNA. Each treatment was conducted three times. *Def* expression was quantified by RT-qPCR. RL32, a ribosomal protein, was used as a reference gene for qPCR. Different letters above standard deviation bars indicate significant differences among means at Type I error = 0.05 (LSD test).

We analyzed for any influence of *SeNOS* expression on AMP expression by suppressing the NO produced from SeNOS using a specific RNAi (Fig 5). A dsRNA specific to *SeNOS* significantly knocked down the *SeNOS* transcript levels (Fig 5A). Under the RNAi conditions, bacterial challenge did not induce AMP expression (Fig 5B). However, adding AA significantly (*P* < 0.05) rescued the AMP expression suppressed by the RNAi treatment.

**Fig 6:**
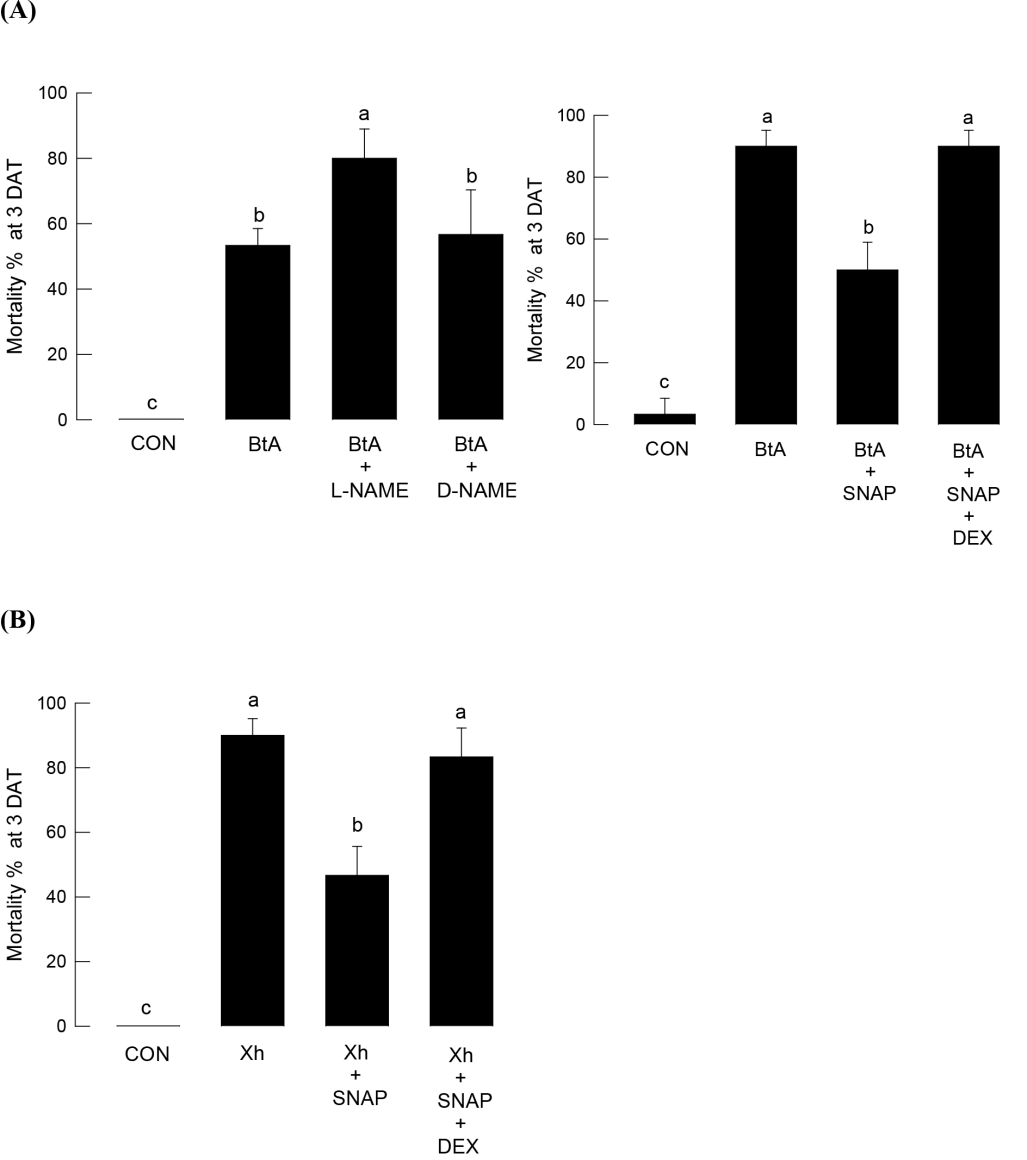
NO/eicosanoid signal against bacterial pathogenicity in *S*. *exigua* fifth instar larvae. (A) Oral pathogenicity using *B*. *thuringiensis aizawai* (BtA). The bacteria were treated by diet-dipping at 7.1 × 10^7^ spores/mL. After 8 h of BtA application, L-NAME (50 μg/larva), D-NAME (50 μg/larva), SNAP (50 μg/larva) or dexamethasone (DEX, 10 μg/larva) were injected. Mortality was measured 72 h after the chemical injection. (B) Hemocoelic infection using *X*. *hominickii* (Xh). The bacteria were injected to larval hemocoel at a dose of 1 × 10^5^ cfu/larva. Chemical treatment used SNAP (50 μg/larva) or DEX (10 μg/larva). Mortality was measured 72 h after the bacterial treatment. Each treatment was conducted three times, and each treatment used 10 larvae. Different letters above standard deviation bars indicate significant differences among means at Type I error = 0.05 (LSD test).

The functional link between NO and eicosanoids in mediating immune response was demonstrated in the bacterial pathogenesis of two entomopathogenic bacteria (Fig 6). The oral toxicity of *B*. *thuringiensis aizawai* was significantly (*P* < 0.05) enhanced by injecting L-NAME, whereas we did not observe the enhanced pathogenicity with D-NAME treatment (Fig 6A); in contrast, SNAP treatment reduced the bacterial pathogenicity. When DEX was added to SNAP treatment, it significantly (*P* < 0.05) inhibited the antibacterial activity induced by SNAP and increased the bacterial pathogenicity. Hemocoelic injection of *X*. *hominickii* was highly potent to *S*. *exigua* larvae (Fig 6B), whereas NO-producing SNAP treatment reduced the bacterial pathogenicity. The suppressed pathogenicity by increasing NO was reversed by adding a PLA_2_ inhibitor.

### 3.3. Toll/IMD pathways are upstream signals of NO/eicosanoids

**Fig 7:**
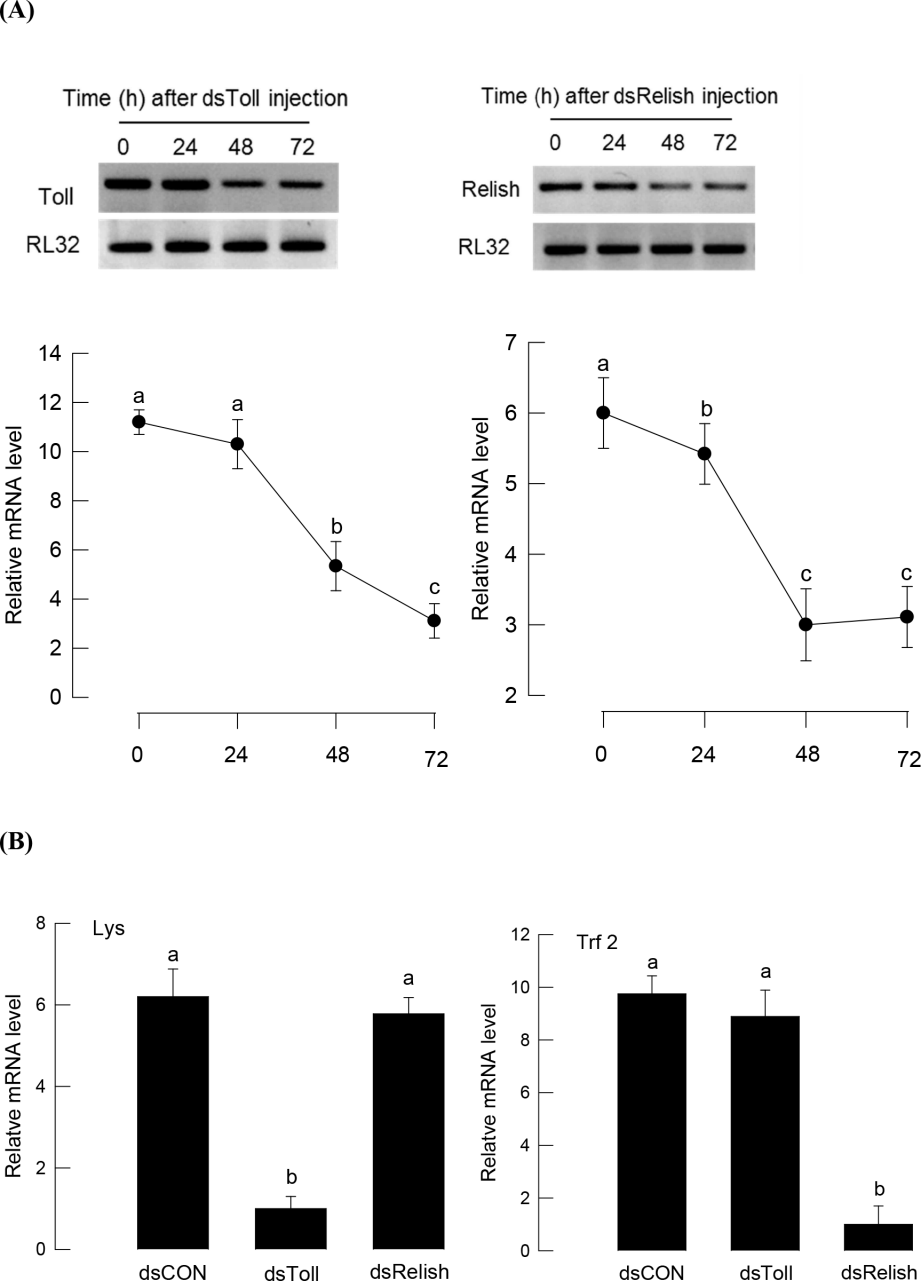
Toll/IMD signaling of *S*. *exigua* and specific AMPs. (A) Specific RNA interference (RNAi) against Toll and IMD signal pathways by injecting 800 ng of dsRNA (dsToll or dsRelish) specific to Toll (contig 06215) or Relish (contig 00977) of *S*. *exigua* transcriptome (SRX259774) to fifth instar larva. Each time point was tested three times. (B) Specific expressional control of Toll/IMD against two AMPs of lysozyme (Lys) and transferrin 2 (Trf 2). After 48 h of dsRNA injection, fat bodies were collected for preparing cDNA. For RNAi control (dsCON), larvae were injected with dsRNA that was specific to a viral gene, *CpBV-ORF302*, in same doses. Each treatment was conducted three times. Target gene (Toll, Relish, Lys, Trf 2) expressions were quantified by RT-qPCR. RL32, a ribosomal protein, was used as a reference gene for qPCR. Different letters above standard deviation bars indicate significant differences among means at Type I error = 0.05 (LSD test).

Toll/IMD signal pathways control AMP gene expression in *S*. *exigua* [39]. To determine any cross-talk of NO with Toll/IMD signals, we inhibited Toll/IMD signals by RNAi and subsequently assessed them for changes in both NO level and PLA_2_ activity. Toll or IMD signals were inhibited by RNAi of *SeToll* receptor or *SeRelish*, respectively (Fig 7A). Under Toll signal RNAi, *lysozyme* (*Lys*) gene expression was significantly suppressed in response to bacterial challenge, but *transferrin 2* (*Trf 2*) gene expression was not. In contrast, under SeRelish RNAi, *Trf2* gene expression was significantly suppressed, but *Lys* gene expression was not (Fig 7B).

**Fig 8:**
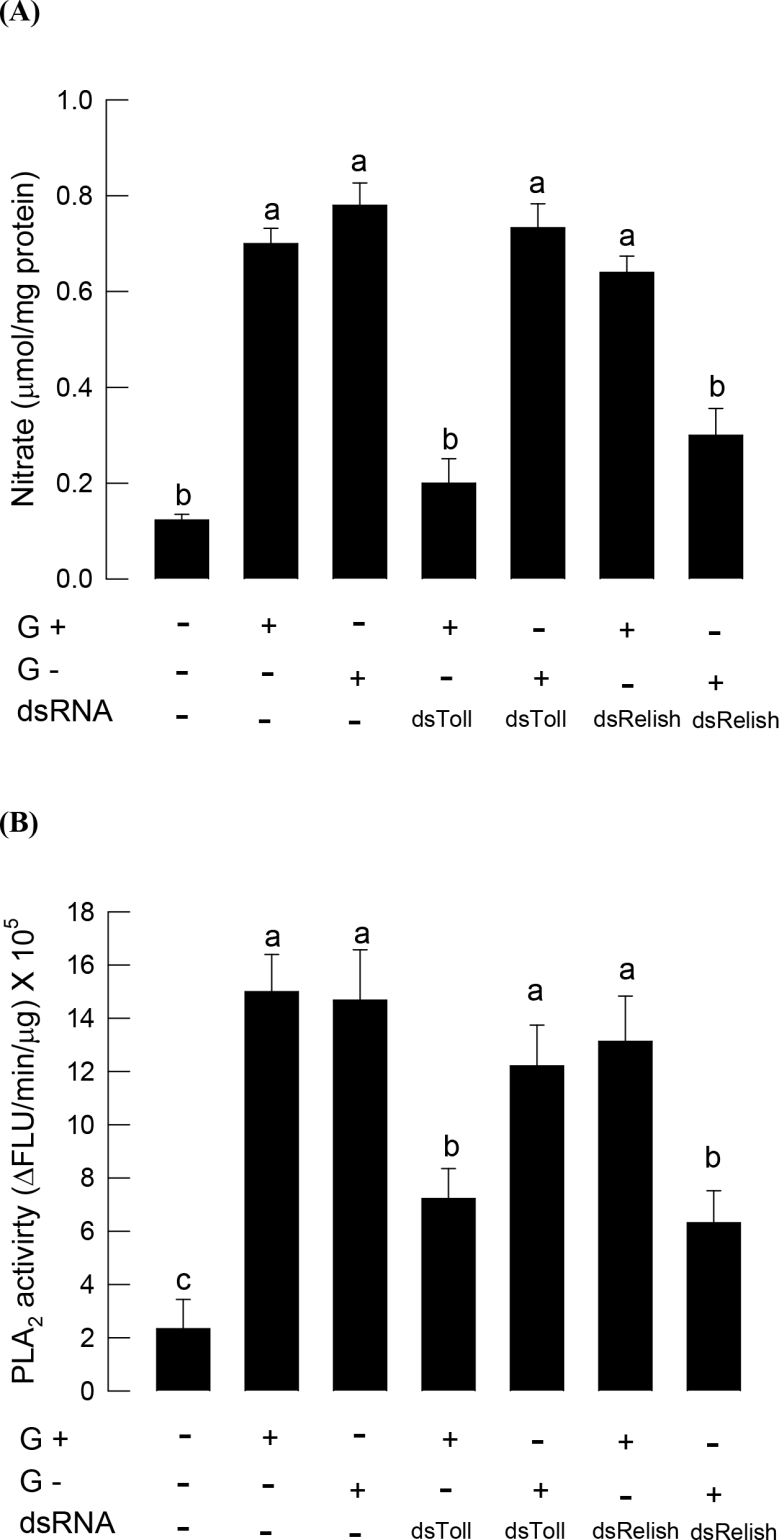
Influence of Toll/IMD signaling on immune mediation by NO/eicosanoid in *S*. *exigua*. Specific RNA interference (RNAi) against Toll and IMD signal pathways was performed by injecting 800 ng of dsRNA (dsToll or dsRelish) specific to Toll (contig 06215) or Relish (contig 00977) of *S*. *exigua* transcriptome (SRX259774) to fifth instar larva. At 48 h after dsRNA injection, immune challenge was initiated by injecting *E*. *coli* for Gram-negative (G-) and *P*. *polymyxa* for Gram-positive (G+) at a dose of 1 × 10^5^ cells per larva. (A) Cross-talk between Toll/IMD and NO signaling. NO signal was quantified by measuring nitrate amount from a whole body after 8 h of bacterial challenge. (B) Cross-talk between Toll/IMD and eicosanoid signaling. Eicosanoid signal was quantified by measuring PLA_2_ enzyme activity after 8 h of bacterial challenge. Each treatment was conducted three times. Different letters above the error bars indicate significant differences between means at Type I error = 0.5 (LSD).

RNAi specific to *SeToll* significantly suppressed NO levels in response to Gram-positive bacterial challenge but not to Gram-negative bacteria (Fig 8A). In contrast, RNAi specific to *SeRelish* suppressed NO levels in response to Gram-negative bacterial challenge but not to Gram-positive bacteria. According to NO level modulated by dsRNA treatments, PLA_2_ activity also changed in a similar pattern (Fig 8B).

**Fig 9:**
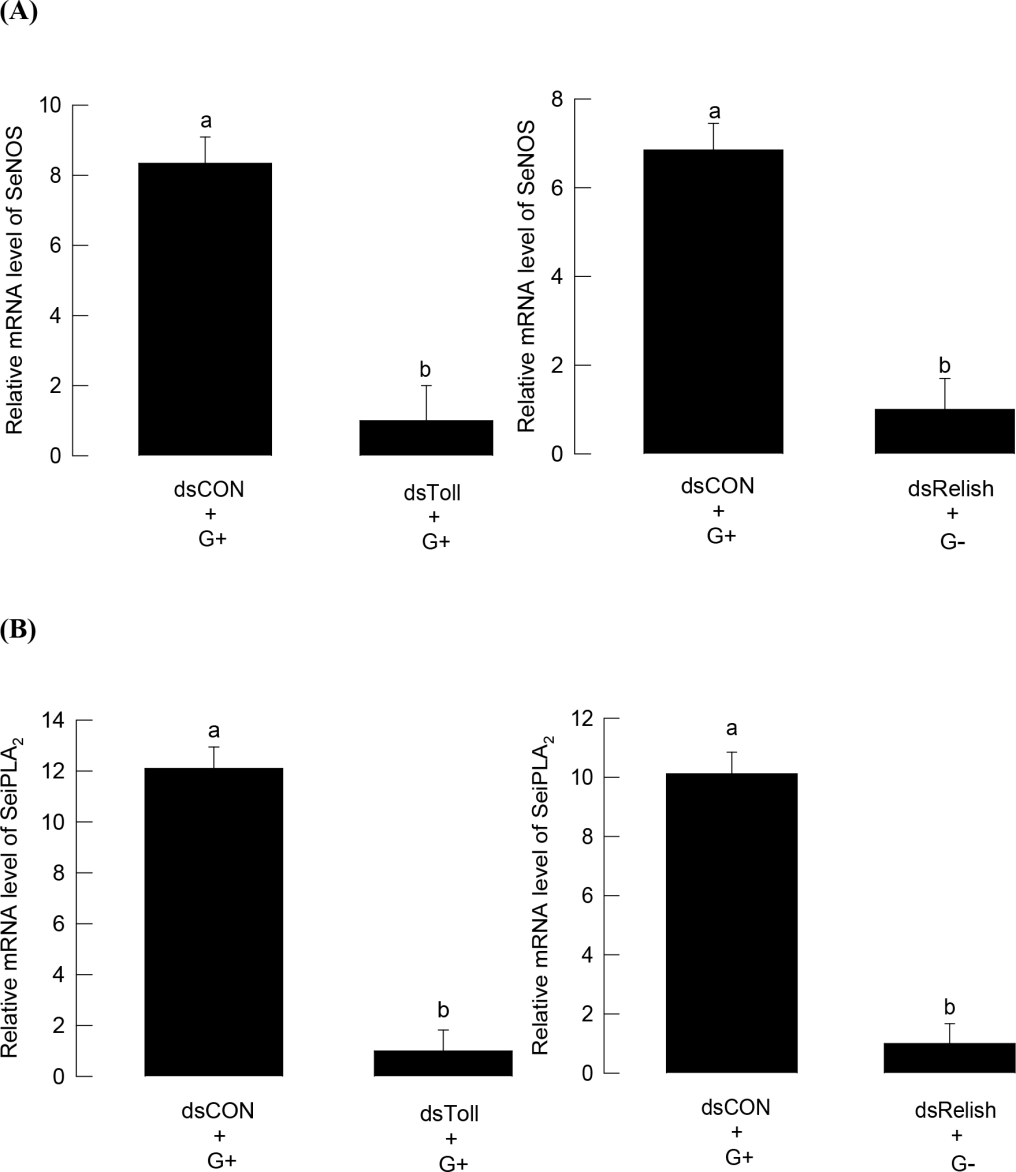
Influence of Toll/IMD signaling on gene expression of (A) NO synthase (SeNOS) and (B) calcium-independent PLA_2_ (SeiPLA_2_) under bacterial challenge in *S*. *exigua*. Specific RNA interference (RNAi) against Toll and IMD signal pathways was initiated by injecting 800 ng of dsRNA (dsToll or dsRelish) specific to Toll (contig 06215) or Relish (contig 00977) of *S*. *exigua* transcriptome (SRX259774) into fifth instar larva. At 48 h after dsRNA injection, immune challenge was initiated by injecting *E*. *coli* for Gram-negative (‘G-’) and *P*. *polymyxa* for Gram-positive (G+) at a dose of 1 × 10^5^ cells per larva. After 8 h of bacterial challenge, fat bodies were collected for cDNA preparation. For RNAi control (dsCON), larvae were injected with dsRNA that was specific to a viral gene, CpBV-ORF302, in same doses. Each treatment was conducted three times. Target gene (SeNOS, SeiPLA_2_) expressions were quantified by RT-qPCR. RL32, a ribosomal protein, was used as a reference gene for qPCR. Different letters above standard deviation bars indicate significant differences among means at Type I error = 0.05 (LSD test).

RNAi treatment of *SeToll* suppressed the inducible expression of *SeNOS* in response to Gram-positive bacterial challenge (Fig 9A), and *SeiPLA*_*2*_*–A* expression was also suppressed (Fig 9B). RNAi treatment of *SeRelish* suppressed the inducible expression of *SeNOS* in response to Gram-negative bacterial challenge, and *SeiPLA*_*2*_*–A* expression was also suppressed.

## Discussion

Both NO and eicosanoids mediate immune responses in *S*. *exigua* and other insects [8]. Our previous study showed that NO mediated a cellular immune response of hemocyte nodule formation by activating PLA_2_ to induce eicosanoid signals [24]. To extend this cross-talk between NO and eicosanoid immune signals in *S*. *exigua*, in this current study, we tested a hypothesis of NO mediation of AMP expression in response to bacterial challenge. The data reported here support our hypothesis that NO signaling cross-talks with eicosanoids, in which NO is an upstream component of eicosanoid signaling in mediating AMP expression in response to the bacterial immune challenge.

NO level was inducible and played an immune-mediating role in AMP gene expression in response to bacterial challenge in *S*. *exigua*. The bacterial challenge increased NO levels approximately fourfold, and we also observed this inducible NO level in our previous study [24]. Moreover, in *M*. *sexta*, bacterial challenge increased NO by approximately tenfold [25]. Because NO is cytotoxic at high concentrations (100–1,000 ×) by rapid increase in mammals [45–47], the relatively mild increase in NO concentration in insects suggests that it plays a role in mediating immune signals to hemocytes and fat body rather than gives a direct toxic effect to pathogens. At low concentrations, NO play a role in mediating cellular and humoral immune responses in mammals [48].

We assessed eight AMPs in this study because their expressions were inducible in *S*. *exigua* in a previous study [39]. Expression of these eight AMPs was inducible in response to Gram-negative bacterial challenge, though four of these AMPs were inducible to Gram-negative bacteria. A NO donor, SNAP, without any bacterial challenge significantly up-regulated the gene expression of all eight AMPs. Furthermore, treatment with L-NAME (a competitive NOS inhibitor) or RNAi against *SeNOS* suppressed AMP gene expression. Our previous study [24] showed that L-NAME completely inhibited the NO level induced by bacterial challenge. Because *SeNOS* is an iNOS in the same way as other lepidopteran *NOS*s [23,25], inhibiting *SeNOS* expression by its specific dsRNA in response to bacterial challenge suggests a shutdown of *de novo* NO synthesis. These results indicate that NO mediates AMP gene expression in response to bacterial challenge. NO induction of AMP gene expression in the absence of bacterial infection was reported in *D*. *melanogaster* [49]. In *B*. *mori*, inducible NO production was responsible for AMP gene expression, in which up-regulation of *NOS* expression was induced by a cytokine [23]. Indeed, regulation of *NOS* expression was directly associated with immune response in *M*. *sexta* [25].

The NO mediation of AMP gene expression was dependent on eicosanoids. Any induction of AMP gene expression by either bacteria or SNAP was suppressed by treatment with an eicosanoid biosynthesis inhibitor. However, adding AA significantly rescued the AMP gene expression. Furthermore, there was a high correlation between NO levels and PLA_2_ activity in response to bacterial challenge. Treatment with dsRNA specific to *SeNOS* suppressed the *SeNOS* expression in the larvae challenged by bacterial infection. These findings suggest that the RNAi treatment prevented the inducible NO production in response to the bacterial challenge. Under this RNAi condition, AA (a catalytic product of PLA_2_) alone significantly rescued the AMP gene expression. Taken together, these results suggest that eicosanoid signaling is downstream of NO mediation to induce AMP gene expression in response to bacterial infection. Because eicosanoids mediate humoral immune reactions [37,38,50,51], we propose that NO mediates humoral as well as cellular immune responses in *S*. *exigua*.

Eicosanoids mediate cellular and humoral immune responses in insects [52]; eicosanoid immune signals act as a common downstream signal for a cytokine and two biogenic monoamines in *S*. *exigua* [18,21]. In addition to what we found in the current study, NO signaling also uses eicosanoids as a downstream signal by activating PLA_2_ activity; the up-regulated PLA_2_ activity, in turn, enhances eicosanoid biosynthesis. The cross-talk between NO and eicosanoids was initially reported from a mouse macrophage cell line, RAW264.7 [53]. In the macrophage cells, lipopolysaccharide treatment induced NOS activity, and the resulting NO activated cyclooxygenase-2 (COX-2), which significantly elevated PG levels. When human fetal fibroblasts stimulated by interleukin 1β were treated with exogenous NO, COX-2 activity was significantly induced [54,55]. Thus, NO interacts with COX-2 to simulate production of pro-inflammatory PGs [56]. In our current study, the increased level of NO activated PLA_2_ activity in *S*. *exigua*, and the reverse direction of cross-talk to increase NO level by eicosanoids is not likely to occur because treatment with PLA_2_ inhibitor did not change NO levels in our previous study [24]. These findings suggest that eicosanoids are a downstream signal of NO to mediate AMP gene expression.

**Fig 10:**
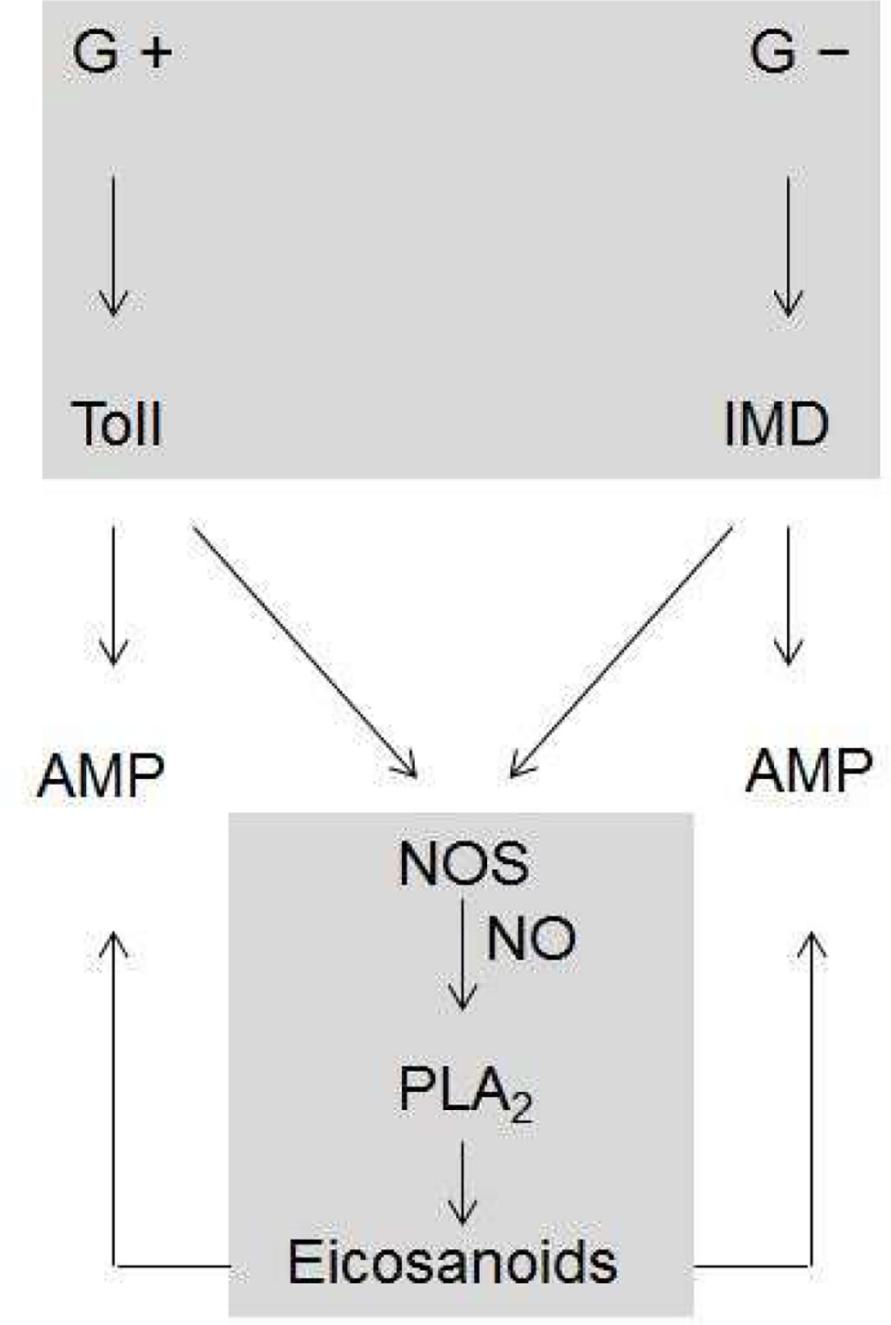
A diagram illustrating cross-talk between Toll/IMD and NO/eicosanoid signal pathways to induce gene expression of antimicrobial peptides in *S*. *exigua*. Gram-negative (G-) and Gram-positive (G+) represent bacterial immune challenges.

AMP gene expression is controlled under Toll/IMD signal pathways in *S*. *exigua* [39]. Through analysis of immune-associated genes on a genome-wide basis, the Toll/IMD immune signals have been demonstrated in several model insects: *Drosophila* [57], *Anopheles gambiae* [58], *Aedes aegypti* [59], *Apis mellifera* [60], *Tribolium castaneum* [61], and *B*. *mori* [62]. Based on a *Drosophila* model, Toll/IMD signal pathways mediate the recognition signals to induce expression of specific AMP genes [1,63]. Toll pathways are activated mainly by lysine-type peptidoglycan of most Gram-positive bacteria and β-1,3-glycan of fungi. The activated Toll receptor recruits a heterotrimeric adaptor (Myd88-Tube-Pelle), which then activates a nuclear translocation of Dif or Dorsal NF-kB transcriptional factor by inactivating Inhibitor kB (IkB) via IkB kinase activity to induce specific AMP genes [64,65]. In contrast, the IMD pathway is activated mainly by diaminopimelic acid-type peptidoglycan of Gram-negative bacteria. Membrane-bound PGRP-LC activates a cytoplasmic death domain-containing adaptor, which results in a proteolytic cleavage of Relish to be translocated into nucleus to induce specific AMPs [66]. A hemocyte transcriptome of *S*. *exigua* provided *SeRelish* and *SeToll* genes, which were confirmed to play crucial roles in mediating the AMP expression signal [39]. A previous work classified *S*. *exigua* AMPs into four groups depending on Toll/IMD signal pathways. *Lysozyme* expression was classified as controlled by the Toll pathway, while *transferrin-2* expression was controlled by the IMD pathway [39]. This current study supported this classification by RNAi treatments. Under this specific RNAi, NO level and PLA_2_ activity were specifically modulated by either Toll or IMD signal pathways. In *D*. *melanogaster*, NO is known to induce cellular and humoral immune responses via Toll/IMD signal pathways [49,67]. Our current study supports the cross-talk between the Toll/IMD signal and NO by inducing *NOS* expression. Furthermore, this current study showed that Toll/IMD signals were specifically activated depending on pathogen type but that both pathways commonly activated NOS to produce NO. The increase in NO in turn activates PLA_2_ activity to synthesize eicosanoids. These findings suggest that Toll/IMD signal pathways are upstream to NO/eicosanoid signaling (Fig 10). Thus the Toll/IMD pathway induction of AMP genes appears to be primary, whereas the NO/eicosanoid signal may be secondary to enhance the AMP gene expression. Activation of PLA_2_ activity by Toll/IMD signal pathways is reported in *T*. *castaneum* [68], in which PLA_2_ activity was induced following bacterial challenge but was inhibited by dsRNAs specific to different Toll and IMD genes. In our current study, immune-associated iPLA_2_-B [69] expression was induced by Toll/IMD pathways. However, it is still unknown how eicosanoids activate AMP gene expression. Stanley et al. [70] showed that PGs application alters gene expression in an insect cell line, suggesting a direct action of eicosanoids to activate AMP gene expression. Alternatively, eicosanoids may activate Toll/IMD pathways to induce AMP gene expression via an autocrine or paracrine mode. Inhibiting eicosanoid biosynthesis using a PLA_2_ mutant line in *D*. *melanogaster* [71] or RNAi of a gene that encoded sPLA_2_ in *Bactrocera dorsalis* [72] suppressed Toll/IMD signal pathways.

In summary, Toll/IMD signal pathways induce NOS expression as well as various AMP genes. The induction of NOS expression by influence of Toll/IMD signal leads to increase of NO concentration, which in turn activates PLA_2_ to synthesize various eicosanoids. These results suggest that eicosanoids are released from immune-activated cells by the elevated NO concentration and activate nearby immune cells including hemocytes and fat body to produce AMPs. Thus, inhibiting eicosanoid biosynthesis results in marked suppression of both cellular and humoral immune responses because eicosanoids mediate downstream signal compared to Toll/IMD and NO signals in *S*. *exigua*.

## Supporting information

S1 Table Primers used for qPCR reactions and dsRNA preparation. (DOCX)
