# Supplementary material for: Nitric oxide mediates antimicrobial peptide gene expression by activating eicosanoid signaling
Source: PLoS One. 2018 Feb 21;13(2):e0193282. doi: 10.1371/journal.pone.0193282 (PMC5821394; doi:10.1371/journal.pone.0193282)
Supplement: S1 Table — (DOCX) [file pone.0193282.s001.docx]

**S1 Table**. Primers used for qPCR reactions and dsRNA preparation

| Genes | Sequences (5′-3′)^1^ | Size (bp) |
| --- | --- | --- |
| Att-1-F | GCTTTCCTCTCCAGGAATAT | 276 |
| Att-1-R | CCTTAGAGTAAATCCAGTGG |  |
| Att-2-F | TCCCGAATGTGCCCAACTTC | 254 |
| Att-2-R | GAAAGATCTGCCGAAAGTAA |  |
| Def -F | ATGGGTGTTAAGGTAATAAA | 303 |
| Def -R | GCAACTACATGTATGACTAA |  |
| Glv-F | CGTGGACATCTTCAGGGCC | 277 |
| Glv -R | GTCGTGTTCAATGCCACC |  |
| Hemo-F | AAGACCAGGGCGAGTACAAG | 347 |
| Hemo-R | AGCGACATGAACCAAGGTTTC |  |
| Lyso-F | ATGCAAAAGCTAACGGTTTTC | 385 |
| Lyso-R | GATTCTTCCATCCATACCAG |  |
| Tf-1-F | GTCCCTCTCTGTCCTGAAGG | 370 |
| Tf-1-R | CAGAAACACGAAGAAAGATG |  |
| Tf-2-F | GATGTTCTGGCGCAGCTGTC | 288 |
| Tf-2-R | CCGGCTGAACGCAAACACAG |  |
| RL32-F | ATGCCCAACATTGGTTACGG | 270 |
| RL32-R | TTCGTTCTCCTGGCTGCGGA |  |
| SeNOS-F | GCGAAGGACCTGTACACTATG | 332 |
| SeNOS-R | CAAGGCCAAACGAGCTTTATG |  |
| SeiPLA_2_-B-RT-F | AGACACTACACTCGGCAAGA | 406 |
| SeiPLA_2_-B-RT-R | CGAGATGGCGCAGTACC |  |
| SeToll-F | GAGTGCGACTGTACAATG | 348 |
| SeToll-R | GGTCGCATCCATCGGTATTC |  |
| SeRelish-F | TGTGATCTAGCAAGTGCATTG | 446 |
| SeRelish-R | ACTTCAATTCCGTCTTCTGTC |  |
| SeNOSRNAi-F | TAATACGACTCACTATAGGGAGAGCGAAGGACCTGTACACTATG | 378 |
| SeNOSRNAi-R | TAATACGACTCACTATAGGGAGACAAGGCCAAACGAGCTTTATG |  |
| SeTollRNAi-F | TAATACGACTCACTATAGGGAGAGAGTGCGACTGTACAATGG | 392 |
| SeTollRNAi-R | TAATACGACTCACTATAGGGAGAGGTCGCATCCATCGGTATTC |  |
| SeRelishRNAi-F | TAATACGACTCACTATAGGGAGATGTGATCTAGCAAGTGCATTG | 490 |
| SeRelishRNAi-R | TAATACGACTCACTATAGGGAGAACTTCAATTCCGTCTTCTGTC |  |
| CpBV-ORF302-F | TAATACGACTCACTATAGGGAGACAATACGGAGCTAGCGTTACG | 466 |
| CpBV-ORF302-R | TAATACGACTCACTATAGGGAGAGGTATGCTTCTCTGCATATGC |  |

^1^ Underlined sequence represents T7 promoter.
